# Cloning and characterization of cDNAs encoding putative CTCFs in the mosquitoes, *Aedes aegypti *and *Anopheles gambiae*

**DOI:** 10.1186/1471-2199-6-16

**Published:** 2005-06-28

**Authors:** Christine E Gray, Craig J Coates

**Affiliations:** 1 Department of Entomology, Texas A&M University, MS 2475, College Station, TX 77843-2475 USA; 2 Interdisciplinary Graduate Program in Genetics, Texas A&M University, MS 2475, College Station, TX 77843-2475 USA

## Abstract

**Background:**

One of the many ascribed functions of CCCTC-binding factor (CTCF) in vertebrates is insulation of genes via enhancer-blocking. Insulation allows genes to be shielded from "cross-talk" with neighboring regulatory elements. As such, endogenous insulator sequences would be valuable elements to enable stable transgene expression. Recently, CTCF joined Su(Hw), Zw5, BEAF32 and GAGA factor as a protein associated with insulator activity in the fruitfly, *Drosophila melanogaster*. To date, no known insulators have been described in mosquitoes.

**Results:**

We have identified and characterized putative CTCF homologs in the medically-important mosquitoes, *Aedes aegypti *and *Anopheles gambiae*. These genes encode polypeptides with eleven C2H2 zinc fingers that show significant similarity to those of vertebrate CTCFs, despite at least 500 million years of divergence. The mosquito CTCFs are constitutively expressed and are upregulated in early embryos and in the ovaries of blood-fed females. We have uncovered significant bioinformatics evidence that CTCF is widespread, at least among *Drosophila *species. Finally, we show that the *An. gambiae *CTCF binds two known insulator sequences.

**Conclusion:**

Mosquito CTCFs are likely orthologous to the widely-characterized vertebrate CTCFs and potentially also serve an insulating function. As such, CTCF may provide a powerful tool for improving transgene expression in these mosquitoes through the identification of endogenous binding sites.

## Background

CTCF (CCCTC-binding factor) was originally identified as a transcriptional repressor in studies of the chicken lysozyme silencer [1] and the regulation of the chicken c-myc gene [2]. Since that time, CTCF has been extensively characterized in vertebrates as a ubiquitously-expressed, highly-conserved, multivalent transcription factor that utilizes different zinc finger (ZF) combinations to specifically bind diverse nucleotide sequences, resulting in the repression or activation of target genes, creation of hormone-responsive silencers and the formation of enhancer-blocking boundary elements (reviewed in [3]). Multiple, independent studies have established vertebrate CTCF as a central player in the regulation of gene expression via its association with every known vertebrate insulator [3-5]. Further characterization of these proteins revealed their insulator function to be central in three contexts: (a) constitutive insulation of the chicken *β*-globin gene at the 5'HS4 site [6,7] and the human apolipoprotein B gene at the 5' boundary [8], (b) imprinted insulation via methylation-sensitive binding to the *Igfr2-H19 *control locus [6,9-14], the *DM1 *locus [5] and the *DLK1/GTL2 *locus [15], and (c) as part of a more complex, multipartite insulator regulated by ligand binding [16]. Most recently, CTCF-dependent insulators have been identified in transitional chromatin, with high levels of H3 acetylation and essentially no CpG methylation, between escape and inactivated genes on both mouse and human inactivated X chromosomes [17]. Finally, *Tsix *and CTCF have been proposed to comprise a regulated epigenetic switch for X-inactivation in mammals [18]. Clearly, CTCF plays a pivotal role at multiple levels of gene regulation and genome organization in vertebrate organisms.

Long thought to be exclusive to vertebrates, a CTCF orthologue was recently characterized in *Drosophila melanogaster *with domain structure, binding site specificity and transcriptional repressor activity similar to that of vertebrate CTCF [19]. Significantly, these researchers also demonstrated that a known *Drosophila *insulator, *Fab8*, mediates enhancer-blocking via CTCF in both *Drosophila *and vertebrate cell lines. We have cloned and characterized two mosquito CTCF-like cDNAs encoding polypeptides with significant similarity and insulator binding properties to both the vertebrate and *Drosophila *CTCFs. Analysis of available genome sequence from numerous invertebrate species yields promising candidates for additional CTCF orthologues. Clearly, this versatile protein has much more ancient roots than once thought.

## Results

### Cloning of *Ae. aegypti *and *An. gambiae *CTCF-like cDNAs

**Figure 1:**
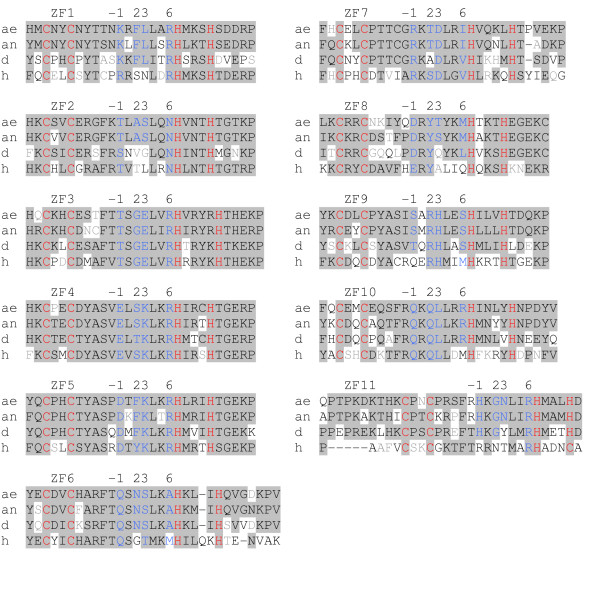
**The zinc-finger (ZF) domain is highly conserved between humans and the dipteran insects, *Ae. aegypti, An. gambiae and D. melanogaster*. **Each of the eleven ZFs were aligned using the ClustalW algorithm. Identical and highly conserved residues are highlighted in gray. Weakly conserved residues, the zinc-coordinating residues, and the amino acids with identical binding site recognition properties are indicated in gray, red and blue font respectively.

A BLAST search using the human CTCF protein sequence [20] as a query uncovered a cDNA from *D. melanogaster *[AAL78208], subsequently characterized by Moon *et al*. [19] as an orthologous CTCF factor. This sequence was then used to query the *An. gambiae *genome assembly at the Ensembl database [21], resulting in a highly significant hit of the predicted novel gene ENSANGG00000015222 (e-139). These two dipteran sequences were aligned with known vertebrate CTCF sequences from *Gallus gallus *[22], *Mus musculus *and *Homo sapiens *[20], *Rattus norvegicus *[NP_114012.1] and *Xenopus laevis *[23] using the ClustalW algorithm (Vector NTI™ Suite 8, InforMax, Inc., 1999). This multiple sequence alignment was used for degenerate PCR primer design. Degenerate PCR amplification, using *Ae. aegypti *larval cDNA as a template, yielded a single PCR product of 504 base pairs, corresponding to a 168 amino acid polypeptide containing six of the eleven predicted zinc-finger domains. PCR amplification was initially performed with an *An. gambiae *larval cDNA template and primers corresponding to the 5' and 3' ends of the predicted novel coding sequence. This yielded a single product of 2040 base pairs, corresponding to a translated polypeptide of 680 amino acid residues. Subsequent 5' and 3' RACE (rapid amplification of cDNA ends) in both species yielded putative full-length cDNAs of 2616 and 4544 base pairs for *Ae. aegypti *(AY935523) and *An. gambiae *(AY939827), respectively. Alignment of the corresponding polypeptide sequences with both the *D. melanogaster *and *H. sapiens *CTCFs revealed significant differences in the N-terminal and C-terminal regions of the protein, however there was 38% identity and 56% similarity across all eleven zinc finger domains (Fig. 1). Furthermore, 68% of the critical binding residues were conserved, despite at least 500 million years of divergence between invertebrate and vertebrate species [24].

### CTCF appears widespread in *Drosophila *species

**Figure 2:**
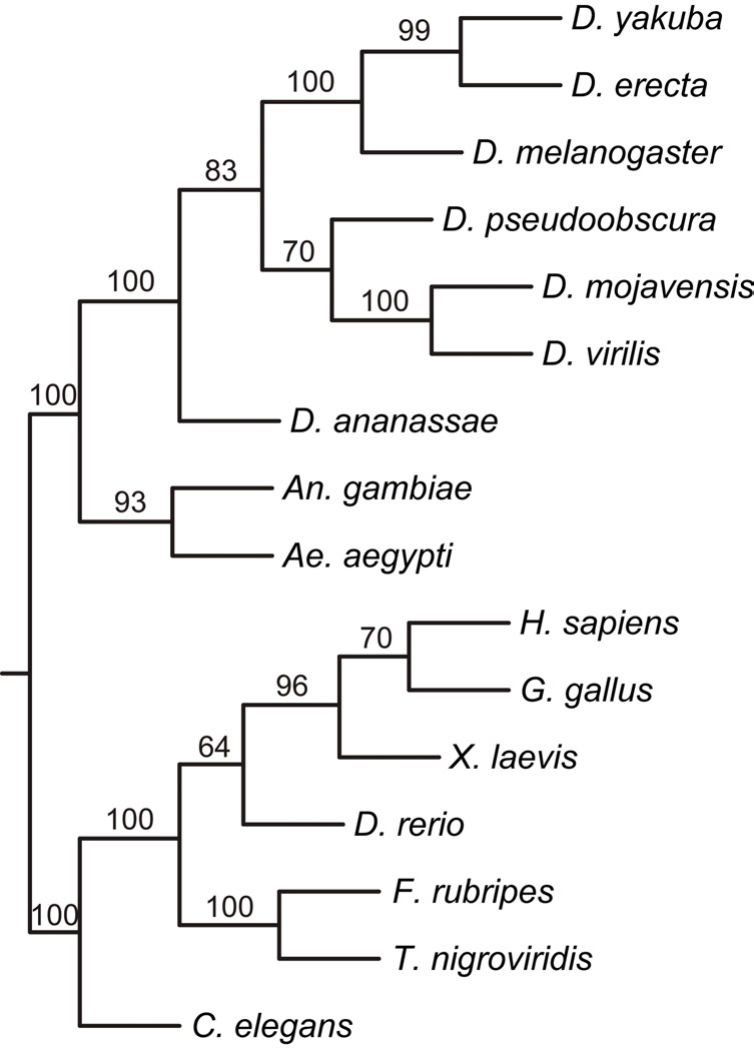
**Phylogenetic analysis of CTCF-like candidates in multiple species. **Dendrogram of a neighbor-joining consensus tree of 5000 bootstrap replicates for an alignment of the 11 ZF region of known and predicted CTCFs. The tree topology is consistent with the taxonomic classification of all *Drosophila *species.

Available genome sequence for multiple drosophilid species was queried at Flybase [25] using the *An. gambiae *amino acid sequence and the tBLASTx algorithm. All species searched produced single hits of very high significance, ≤ e-126. Each of these was submitted as a BLASTp query of the non-redundant database at NCBI [26] and confirmed to be a significant match to known CTCFs. Sequences with complete zinc finger regions were trimmed to the zinc-finger region plus five flanking amino acid residues and aligned with the corresponding region of CTCFs from *H. sapiens*, *G. gallus*, *X. laevis*, *Danio rerio *[NP_001001844], *Tetraodon nigroviridis *[CAF99566], and *Fugu rubripes *(Ensembl novel gene SINFRUG00000147322). The corresponding region of zinc finger protein 2 from *Caenorhabditis elegans *[NP_500033], a protein that contains 11 C2H2 zinc finger domains, a coil-coil region and predicted nuclear localization sequence, was also included in the alignment and used as an outgroup in the subsequent phylogenetic analysis. Two consensus distance-based trees, Neighbor-Joining [27] (Fig. 2) and Fitch-Margoliash [28] (data not shown), were generated with 5000 bootstrap replicates using the Phylip software package [29,30]. Additionally, a maximum-likelihood tree generated by 200,000 iterations of Tree-Puzzle [31] (data not shown) and a Bayesian analysis tree generated by 200,000 cycles of BAMBE [32] with 20,000 cycles of burn-in (data not shown), yielded identical branch topologies.

### Mosquito *CTCF *is expressed constitutively in all developmental stages and is upregulated in early embryos and the ovaries of blood-fed females

**Figure 3:**
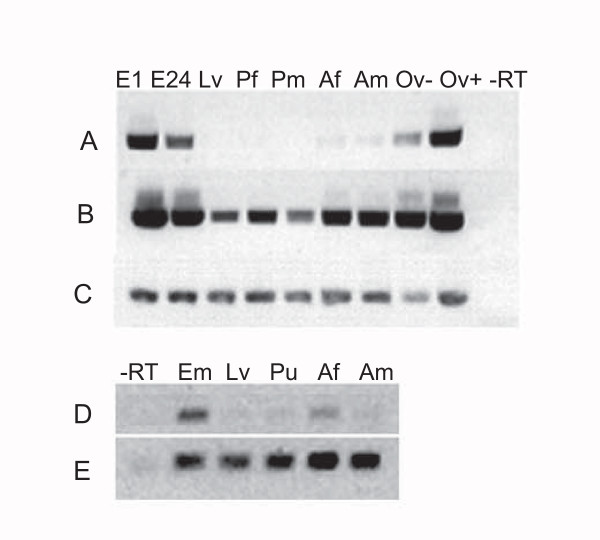
**Developmental expression profile of CTCF protein in *Ae. aegypti and D. melanogaster***. The expression of *CTCF *was analyzed using RNA isolated from multiple individuals at each of the indicated stages: E1 and E24 (embryos ≤ 1 hr and 24 hrs post-oviposition respectively), Lv (larvae), Pf (female pupae), Pm (male pupae), Pu (pupae), Af (adult females), Am (adult males), Ov- and Ov+ (ovaries from non-blood-fed and blood-fed females respectively). -RT, no reverse-transcriptase. A. and B.) *Ae. aegypti CTCF*, 20 cycles and 30 cycles respectively. C.) *Ae. aegypti S17*, 25 cycles. D.) *D. melanogaster CTCF*, 25 cycles. E.) *D. melanogaster Rp49*, 20 cycles.

Reverse-transcriptase (RT)-PCR amplifications of RNA isolated from embryos, ovaries, larvae, pupae and adults shows CTCF expression across all stages of development and in the ovarian tissues of both *Ae. aegypti *and *D. melanogaster *(Fig. 3). Early *Ae. aegypti *embryos and ovarian tissues from both species clearly show increased expression levels.

### Polyclonal antisera raised against *An. gambiae *CTCF recognizes a single protein band in lysates from *An. gambiae *Sua4 cultured cells

**Figure 4:**
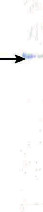
***An. gambiae *CTCF polyclonal antisera recognizes a distinct band migrating ~84 kD in SDS-PAGE. **Lysates from *An. gambiae *Sua4 cultured cells were separated by 8% SDS-PAGE and immunoblotted with CTCF rabbit antisera. The arrow indicates the position of the 81.1 kD marker. The bounding box marks the edges of the gel.

Immunoblotting of total cell lysate from *An. gambiae *Sua4 cultured cells with rabbit antisera raised against a c-terminal fragment of *An. gambiae *CTCF results in identification of a single band migrating at ~84 kD (Fig. 4).

### Mosquito CTCF binds *in-vitro *to both the chicken 5'HS4 and the *Drosophila Fab8 *insulators

**Figure 5:**
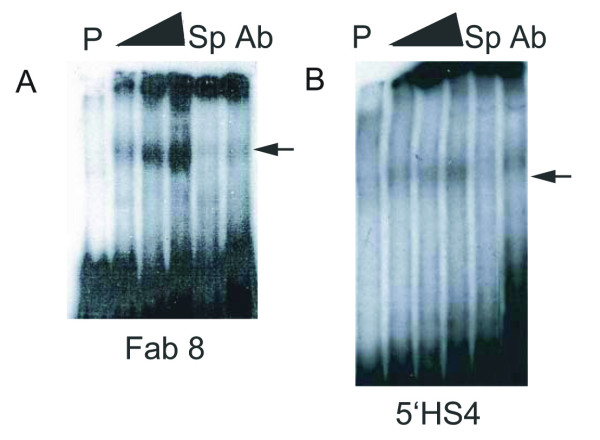
***An. gambiae *CTCF specifically binds the chicken 5'HS4 and Drosophila Fab8 insulator sequences. **Sua4 cells were lysed and increasing amounts of total cell protein (1.5, 7.5, 15 μg represented as solid triangle) were incubated with radiolabeled insulator sequences as follows: A.) *Drosophila Fab8 *insulator sequence [19]; B.) chicken β*-globin *FII insulator sequence [6]. The complex was competed (Cp) with ~150-fold excess of cold, unlabeled probe DNA and supershifted (Ab) with polyclonal antibody sera raised against the C-terminal fragment of *An. gambiae *CTCF. The probe only lane is indicated by P.

As we were unable to express the full-length mosquito CTCF protein in bacteria, whole cell lysates were prepared from the *An. gambiae *Sua4 [33] cell line and used in an electrophoretic mobility shift assay (EMSA) to assess whether mosquito CTCF could bind known CTCF-associated insulator sequences (Fig. 5). The intensity of the shifted bands increased with application of greater amounts of protein lysate. The detectable complex was competed by cold, unlabeled probe, indicating that binding was indeed specific. In addition, all reactions contained a 1200-fold excess of cold, non-specific C/G-rich sequences, further illustrating specificity. Finally, the complex could be partially shifted by polyclonal anti-sera generated against the C-terminal region of the *An. gambiae *CTCF protein.

## Discussion

Vertebrate CTCFs, from fish to human, are ≥ 98% identical across the entire zinc finger core of the protein. Comparison of the three dipteran CTCFs reveals 54% identity and 68% similarity within this same region. In addition, amino acid residues considered critical for DNA binding [34] are 89% conserved among these three insect species. This apparent discrepancy can be partially addressed by investigating the molecular substitution rate heterogeneity among vertebrates and invertebrates. Recent maximum likelihood analysis of a set of 50 nuclear genes for vertebrates and dipterans, with *Arabidopsis *as an outgroup, suggests that the rate of vertebrate molecular evolution slowed considerably with respect to that of dipterans, prior to the origin of the crown-group, Osteichthyes [24]. The much shorter generation times of dipterans have undoubtedly facilitated significant differences in their genome sizes (ranging from 179 Mb in *D. melanogaster *[35] to 813 Mb in *Ae. aegypti *[36]) and gene organization patterns, attributable primarily to the number and distribution of repetitive sequences [37]. This would perhaps result in predictions of even greater sequence divergence than is observed in the CTCF genes. It seems likely that at least some of the many attributed vertebrate functions of CTCF are ancestral.

Each of the species examined yielded a single, extremely significant match followed by numerous matches of lesser significance, suggesting a single copy locus. Significant divergence in available N-terminal or C-terminal sequence supports the earlier observation that dipteran genomes have evolved very quickly, and thus these regions may not be critical to the conserved ancestral function(s) of this gene. Additionally, these regions may be more directly involved in protein-protein interactions with other proteins having likewise undergone evolutionary adaptation. High bootstrap support and essentially identical trees generated by four independent methods establishes the tree presented in Fig. 2 as representative of the evolution of this gene sequence. Less bootstrap support in the vertebrate clade is more indicative of the homogeneity of the sequence, rather than uncertainty as to where these species should be located in the tree. Clearly, CTCF is present in vertebrates from fish through mammals and is highly conserved. Of interest is its consistent presence in all *Drosophila *species queried. The relatedness of the protein sequences mirror the accepted taxonomic relationships among these species as presented at FlyBase [25], likely indicative of a conserved critical function. Significant EST evidence from the flour beetle, *Tribolium castaneum*, the honey bee, *Apis mellifera*, and the silkworm moth, *Bombyx mori*, suggests the presence of CTCF-like genes in multiple insect orders.

The RT-PCR data from both mosquito and fly are consistent with one another, repeatable, and in agreement with both *in-situ *hybridization data [38] posted for the fly at the Berkeley Drosophila Genome Project website [39] and fly microarray data summarized at Yale University's Drosophila Developmental Gene Expression Timecourse website [40]. *In-situ *hybridization shows high-levels of *Drosophila CTCF *transcript ubiquitously distributed throughout stage 1–3 embryos. mRNA levels then decrease until approximately stage 9 where they then increase primarily in the developing nervous and sensory tissues. The neural-specific expression pattern also corresponds to findings in *X. laevis *where *in-situ *hybridization with staged embryos revealed weak homogeneous staining prior to stage 14, with subsequent upregulation in neural tissues and the sensory organs of the head [23]. Furthermore, over-expression of CTCF in mice during early embryogenesis resulted in decreased expression of the highly conserved homeobox gene *Pax6*, causing ocular defects [34]. Microarray data analysis clusters fly CTCF (CG8591) with genes exhibiting a single peak in expression during development, those showing significant expression increases in early embryogenesis, genes with expression changes of at least four-fold across development, and those expressed in the female germline [41]. Taken together, these expression data and the corresponding functional data from vertebrates suggest that CTCF may indeed also be multi-functional in insects. Some possible roles include the regulation of homeobox genes like *Pax6*, the facilitation of chromatin organization during early development and the establishment and/or maintenance of heterochromatic and euchromatic regions.

The EMSA data support a role for CTCF in endogenous mosquito insulator function and confirm recent findings that the insulator function of CTCF is conserved from invertebrate to vertebrate species [19]. Currently, position effect and position-effect variegation complicate efforts to establish stable transgenic lines in *Ae. aegypti *and other mosquitoes. Particularly problematic is the highly repetitive nature of much of the intergenic sequence, as well as the compact nature of the genome, which places regulatory elements from neighboring genes in close proximity to one another, where they may inappropriately impact the transgene of interest. The ability to flank transgenes with short, conserved endogenous insulator sequences could significantly improve observed expression levels, and possibly increase the frequency of recovery of transgenic individuals.

## Conclusion

We have cloned the cDNAs for two putative mosquito CTCF proteins. We have presented bioinformatics evidence that CTCF is likely present in many arthropod species and that the ancestral portion of the protein is clearly the zinc-finger region. Constitutively expressed in all life stages, mosquito CTCFs are highly upregulated in early embryos and in the ovarian tissues of blood-fed female mosquitoes. Finally, mosquito CTCF specifically binds both the chicken 5'HS4 *β-globin *and the fly *Fab8 *insulator sequences. Further characterization of these CTCFs and their binding sites will provide a promising avenue for insulating transgenes in these medically-important mosquito species.

## Methods

### Isolation of RNA and preparation of cDNA by reverse-transcription

Total RNA was isolated from ~30 mg each of *Ae. aegypti *and *An. gambiae *larvae using the RNeasy^® ^Mini Kit (Qiagen, Valencia, CA) followed by DNase I-treatment with DNA-free™ (Ambion, Austin, TX) and was used to synthesize first strand cDNA using the SuperScript II™ reverse transcriptase (Invitrogen, Carlsbad, CA) following the manufacturer's instructions. In order to increase the efficiency of the reverse-transcription reaction, 150 ng/μL of T4 Gene 32 Protein [42] was added to the 1^st ^strand buffer.

### Isolation of *Ae. aegypti *CTCF by degenerate PCR amplification

The amino acid sequences of all known and predicted CTCFs [EAA11339.1, AAL78208, AAG40852, NP_031820, NP_114012, P49711 and Q08705] were identified using the BLAST search algorithm at the National Center for Biotechnology Information (NCBI) website  and aligned using the ClustalW algorithm in the Vector NTI™ Suite (InforMax, Inc., 1999). Two completely nested and degenerate PCR primer pairs were designed to a highly-conserved 168 amino acid region using CODEHOP [43,44]. A 504 base pair nested PCR product was obtained from *Ae. aegypti *larval cDNA using G-1F and K-1R primers in the first PCR reaction, followed by a nested reaction with primers G-2F and J-1R. Each reaction was performed with 2 mM MgCl_2_, 0.2 μM each primer, 10 mM dNTPs, 0.5 μL cDNA or 1st reaction product and 2.5 units of Taq polymerase (Continental Lab Products, San Diego, CA). The following touchdown PCR conditions were used: 96°C for 4'; 2 cycles of 96°C for 20", 72°C for 1'; 11 cycles of 96°C for 20", 71°C -1.0°C/cycle for 15", 72°C for 45"; 25 cycles of 96°C for 15", 59°C for 15", 72°C for 45"; final extension at 72°C for 2'. Degenerate primers were as follows: G-1F 5' cattccgaggacccgccncayaartg 3', G-2F 5' ggccgctgcagaaccacctiaayacncaya 3', J-1R 5' cgcactgctcgcacctgwancayttytc 3', K-1R 5' ccaggtccagcagctgcykytgickraa 3'.

### PCR-amplification and cloning of *An. gambiae CTCF*

The predicted ORF of *An. gambiae *CTCF was PCR amplified from ~100 ng of cDNA with 0.2 μM of each primer and 2.5 units of Herculase^® ^Hotstart DNA Polymerase (Stratagene, La Jolla, CA) per the manufacturer's instructions using the following conditions: 95°C for 2'; 5 cycles of 95°C for 30", 55°C for 30", 72°C for 2'45"; 25 cycles of 95°C for 30", 65°C for 30", 72°C for 2'45"; final extension at 72°C for 5'. The primer sequences were: *Anopheles*CTCFforw 5' caaacgccatatggaggacgtggagctgatat 3' and *Anopheles*CTCFrev 5' attacctcttgcggccgcttccgtggagaggataaact 3'.

### Rapid amplification of cDNA ends (RACE) in *Ae. aegypti *and *An. gambiae*

Total RNA was prepared from freshly collected and snap-frozen larvae using the RNeasy^® ^Mini Kit (Qiagen) and immediately DNase I-treated with DNA-free™ (Ambion) according to the manufacturers' instructions. The BD SMART™ RACE cDNA Amplification Kit (Clontech, Palo Alto, CA) was then used to prepare first-strand cDNA and to amplify 5' and 3' RACE products according to the manufacturer's instructions. The gene-specific primers (GSPs) used for each species were: *Aedes*GSP1 5' gtctgtcttgcgcccacatgttg 3', *Aedes*GSP2 5' cgaaagcacgtttacaacttctgg 3', *Anopheles*GSP1 5' ccacaggtcgtcgggcagagtttgca 3', *Anopheles *GSP2 5' caatcggagtaagattgtccgaagaaaggtct 3'. GSP1 indicates the primer used for 5' RACE reactions while GSP2 indicates the primer used for 3' RACE reactions. Reaction conditions were as follows: 94°C for 5'; 5 cycles of 94°C for 10", 72°C for 3'; 5 cycles of 94°C for 10", 70°C for 10", 72°C for 3'; 25 cycles of 94°C for 10", 68°C for 10", 72°C for 3'; final extension at 72°C for 8'.

### Cloning and sequencing of PCR and RACE products

Products were visualized on a 1% agarose gel, gel purified, cloned into pGEM-T (Promega, Madison, WI) and had their DNA sequence determined using an ABI 3100 capillary sequencer with M13 (-20) and M13 Reverse primers followed by primer walking. At least 3 different clones were analyzed for each PCR or RACE product. The resulting sequences have been deposited in the NCBI GenBank database and have the following accession numbers: [AY935523] (*Ae. aegypti*) and [AY939827] (*An. gambiae*).

### Phylogenetic analysis

Sequences were trimmed to the 11 ZF region plus five flanking amino acid residues and aligned using MultAlin [45] with the Blosum62 model, a gap opening penalty of 35, a gap extension penalty of 0.5 and no end gap penalty. The resulting alignment was analyzed using the Phylip software package [29]: bootstrapped (5000 replicates) with Seqboot, a distance matrix computed using Protdist (5000 datasets), the matrix submitted to Neighbor or Fitch (5000 trees), a consensus tree determined using Consense and the tree drawn using Drawgram. The MultAlin alignment was also submitted to Tree-Puzzle [31] with 200,000 replicates and to BAMBE [32] with 200,000 cycles and 20,000 burn-in.

### Reverse-Transcriptase (RT)-PCR analysis/developmental profile

Total RNA was prepared from freshly collected and snap-frozen samples, DNase treated and the reverse-transcription reaction performed as described above. PCR reactions were assembled with 100 ng cDNA template, 10X buffer, 1.5 μL 10 mM dNTPs, 0.2 μM each primer (Table 1) and 1 μL Advantage2 Taq Polymerase (Clontech) in a total volume of 50 μL. Reaction conditions were as follows: 95°C for 5'; 20, 25 or 30 cycles (see Fig. 3) of 95°C for 15", 55°C for 15", 72°C for 30"; final extension at 72°C for 2'. Products were electrophoresed on a 2% agarose gel, stained with ethidium bromide, destained with ddH_2_O and imaged. The constitutively expressed *D. melanogaster Rp49 *gene (153 bp product) and *Ae. aegypti S17 *gene (200 bp product) were used as controls. Primers were as follows: *Aedes*RT-Forw 5' gtgtttcattgcgagctttgcc 3', *Aedes*RT-Rev 5' tgtctcgatcctccggaatg 3', *S17*RT-Forw 5' cgaagcccctgcgcaacaagat 3', *S17*RT-Rev 5' cagctgcttcaacatctccttg 3', *Drosophila*RT-Forw 5' atggagactcacgatgattcgg 3', *Drosophila*RT-Rev 5' ctcgtcgccattaaccagct 3', *Rp49*RT-Forw 5' gcgcaccaaggacttcatc 3', *Rp49*RT-Rev 5' gaccgactctgttgtcgatacc 3'.

### Generation of polyclonal antisera against *An. gambiae *CTCF

The coding sequence for a C-terminal region (amino acid residues 444–680) was PCR amplified and cloned into the pET-30 plasmid (Novagen, VWR International, Bristol, CT), expressed in *E. coli *(BL21-DE3) and His-tag purified on a Ni-NTA column (Novagen). The purified protein was used to immunize two New Zealand white rabbits following standard procedures.

### Immunoblotting

Sua4 cells were lysed in ice-cold lysis buffer (50 mM Tris, pH 7.8; 150 mM NaCl; 1% IGEPAL CA360 (Sigma, St. Louis, MO)) with Complete Protease Inhibitor Cocktail (Roche, Indianapolis, IN) and 1 mM PMSF. Total cell lysate protein was quantitated using the BCA Protein Assay (Pierce, Rockford, IL), aliquoted and frozen at -20°C. Total cell lysate was separated on 8% SDS-PAGE gel and electroblotted to a PVDF membrane in 1X Towbin buffer according to standard protocols. Upon completion of the protein transfer, the gel was washed twice for 10 minutes in 1X TBS buffer (10 mM Tris-HCl, pH 7.5; 150 mM NaCl). It was then blocked in blocking buffer (1.5% non-fat dry milk (NFDM), 1.5% fraction V Bovine Serum Albumin (BSA), 1X TBS, 0.05% Tween-20) with 20% 5X casein (Novagen), in a sealed bag overnight at 4°C. The blot was then washed twice for 10 minutes in 1X TBSTT and once for 10 minutes in 1X TBS and was incubated for 1 hour at room temperature on an orbital shaker with CTCF polyclonal antisera diluted 1:250 in blocking buffer without casein. After antibody binding, the blot was washed twice in 1X TBSTT (1X TBS, 0.05% Tween-20, 0.2% Triton X-100) for 10 minutes and once in 1X TBS for 10 minutes. Anti-Rabbit IgG (Fc) AP conjugate (Promega, Madison, WI 53711) was diluted 1:7500 in blocking buffer without casein and incubated with the blot for 1 hour at room temperature on an orbital shaker. The blot was then washed for 10 minutes five times in 1X TBSTT. Finally, it was developed for 1–10 minutes in Sigma-FAST™ (Sigma Aldrich Chemical Company, St. Louis, MO 63178) according to the manufacturer's instructions.

### Electromobility Shift Assay (EMSA)

Sua4 cell lysates were prepared and the total protein quantitated as described above. Probes for EMSA were amplified and simultaneously labelled with ^α-32^P (Amersham) by PCR using the following primers: 5'HS4Forw 5' gagctcacggggacagcccccc 3', 5'HS4Rev 5' aagctttttccccgtatccccc 3', Fab8Forw 5' ggcacaatcaagttaatgttgg 3', Fab8Rev 5' gcaagcgaagagttccattc 3'. The chicken 5'HS4 fragment (250 bp) was amplified from pJC13-1 [46] and the *Drosophila Fab8 *fragment (309 bp) was amplified from *Drosophila *genomic DNA. The binding reaction protocol was adapted from Filippova *et al*. [20]. Approximately 10 fmol of labelled probe was incubated for 15 minutes on ice with 0, 1.5, 7.5 or 15 μg of total cell protein in binding buffer (1X PBS with 5 mM MgCl_2_, 0.1 mM ZnSO_4_, 1 mM DTT, 0.1% IGEPAL CA360 (Sigma), 10% glycerol) in the presence of a mixture of non-specific, cold, double-stranded competitor DNAs (500 ng polydI· polydC, 500 ng polydG· polydC, 500 ng SpI oligos, 500 ng Egr1 oligos). The SpI and Egr1 ds oligos contain strong, C/G-rich binding sites for the zinc-finger proteins SpI and Egr1 respectively. Sample 5 contained 150-fold excess unlabeled specific competitor. For the supershift, anti-sera against the *An. gambiae *CTCF was then added and the reactions incubated an additional 15 minutes on ice. Complexes were separated from the free probe on a 5% native PAGE gel in 0.5X TBE. The gel was run for 3.5 hours at 4°C at 10 V/cm.

## Authors' contributions

CEG carried out the studies described in this paper and drafted the manuscript. CJC participated in the design and planning of this study and edited the manuscript. Both authors conceived of the study and have read and approved the final manuscript.
